# Analysis of miRNA‐mRNA regulatory network revealed key genes induced by aflatoxin B1 exposure in primary human hepatocytes

**DOI:** 10.1002/mgg3.971

**Published:** 2019-09-09

**Authors:** Ziyang Zhang, Dongyang Tang, Bin Wang, Zhiwei Wang, Mingjiu Liu

**Affiliations:** ^1^ School of Life Science and Technology Henan Institute of Science and Technology Xinxiang P.R. China; ^2^ Collaborative Innovation Center of Modern Biological Breeding Henan Institute of Science and Technology Xinxiang P.R. China; ^3^ Department of Experimental Management Center Henan Institute of Science and Technology Xinxiang P.R. China

**Keywords:** aflatoxin B1, hepatocellular carcinoma, miRNA/mRNA regulatory network

## Abstract

**Background:**

Aflatoxin B1 (AFB1) exposure is a crucial factor to initiate hepatocellular carcinoma (HCC). However, comprehensive microRNA (miRNA)‐message RNA (mRNA) regulatory network regarding AFB1‐associated HCC is still lacking. This work was aimed to identify miRNA‐mRNA network in primary human hepatocytes after AFB1 exposure.

**Methods:**

A miRNA expression dataset GSE71540 obtained from the gene expression omnibus (GEO) was used to identify differentially expressed miRNAs (DEMs) after AFB1 exposure using GEO2R. Target genes of these DEMs were identified using TargetScan V_7.2, miRDB, PITA, miRanda, and miRTarBase. Gene ontology (GO) annotation and Kyoto Encyclopedia of Genes and Genomes (KEGG) enrichment analyses were performed at Database for Annotation, Visualization and Integrated Discovery (DAVID). miRNA‐mRNA regulatory network was established by analyzing three enriched KEGG pathways significantly correlated with HCC onset and then visualized at CytoScape.

**Results:**

In this work, nine upregulated and nine downregulated DEMs were identified. Functional enrichment analyses showed that these predicted target genes were significantly associated with cancer development. Analysis of three enriched pathways related to the onset of HCC identified 13 and nine target genes for upregulated DEMs and downregulated DEMs, respectively. Subsequently, the miRNA‐mRNA regulatory networks were constructed.

**Conclusions:**

In conclusion, miRNA‐mRNA regulatory network was established, which will help to understand the mechanism underlying the AFB1‐induced onset of HCC.

## 1 INTRODUCTION

Liver cancer ranks the fourth place of all newly identified cancer cases but the third place of cancer‐related deaths in China (Chen et al., 2016). Hepatocellular carcinoma (HCC) alone is reported to represent about 75%–85% of all liver cancer cases (Bray et al., 2018). Exposure to aflatoxin B1 (AFB1) is considered as a trigger for the initiation of HCC (Llovet et al., 2016). However, genes alteration in primary human hepatocytes after AFB1 exposure remains to be elucidated.

Aflatoxin B1 is believed to be transformed to AFB1‐8,9‐epoxide by a specific P450 enzyme and then interact with cellular proteins or DNA to increase the risk of developing into HCC (Groopman, Kensler, & Wild, 2008). It has also been recognized that AFB1 exposure will cause persistent epigenetic changes (Ferreira et al., 2019; Rieswijk et al., 2016). For example, the mutation of *P53* (191170), a well‐characterized tumor suppressor gene, is often found in tissues or cells encountered with AFB1, which will lead to the loss function of *P53* (Shen & Ong, 1996). Moreover, it was found that exposure of AFB1 will cause aberrant methylation status in genes including *RUNT RELATED TRANSCRIPTION FACTOR 3* (600210), *LONG INTERSPERSED NUCLEAR ELEMENT 1* (151626), and so on to initiate carcinogenesis (Wang et al., 2017). In addition, specifically altered microRNA (miRNA) including *MIR‐4651* and *MIR‐34a* (611172) have been observed in AFB1‐induced hepatotoxicity (Wu et al., 2017; Zhu et al., 2015). However, genes altered after AFB1 exposure in human hepatocytes remain to be elucidated.

MicroRNAs are endogenous noncoding RNAs with the length of 18–25 nucleotides, which can directly regulate gene expression through 3’‐untranslated region binding (Bartel, 2009). It has been reported that miRNAs are crucial modulators for various cellular behaviors including cell growth, invasion, apoptosis, and so on (Gandellini, Doldi, & Zaffaroni, 2017; Jafri, Al‐Qahtani & Shay, 2017). Studies focusing on the investigations of abnormally expressed miRNAs in human cancers revealed that miRNA has crucial roles in tumorigenesis (Gandellini et al., 2017; Jafri et al., 2017).

Here, we are interested to investigate abnormally expressed miRNAs after AFB1 exposure with the aim to understand key miRNAs that can trigger HCC carcinogenesis. Therefore, in this study, we investigated dysregulated miRNAs in human hepatocytes treated with AFB1. Finally, novel miRNA‐message RNA (mRNA) regulatory networks associated with HCC onset were constructed.

## 2 MATERIALS AND METHODS

### 2.1 Microarray dataset

GSE71540, obtained from Gene Expression Omnibus (https://www.ncbi.nlm.nih.gov/geo/query/acc.cgi?acc=GSE71540), is a miRNA expression profile dataset submitted by Linda Rieswijk. This dataset contained data from primary human hepatocytes subjected to 1 µM of AFB1 for 5 days followed by a 3 days wash‐out period or not.

### 2.2 Identification of differentially expressed miRNAs

GEO2R, an algorithm based on R language limma package, was employed to analyze differentially expressed miRNAs (DEMs) in this dataset. Cut‐off criteria were set as *p* < .05 and |log2 fold‐change| ≥ 1.5. Volcano plot was established using the plug‐in tool at SangerBox (http://sangerbox.com) to help us identify the overlapping genes. Heatmap was also built by plug‐in tool at SangerBox (http://sangerbox.com) to give a direct presentation of the expression status of genes in different samples.

### 2.3 Identification of target genes for DEMs

Targets of these identified DEMs were predicted at five miRNA target prediction websites including TargetScan V_7.2 (http://www.targetscan.org/vert_72/, Agarwal, Bell, Nam, & Bartel, 2015), miRDB (http://www.mirdb.org/miRDB/index.html, Liu & Wang, 2019), PITA (https://genie.weizmann.ac.il/pubs/mir07/mir07_data.html, Kertesz, Iovino, Unnerstall, Gaul, & Segal, 2007), miRanda (http://www.microrna.org/microrna/home.do, Betel, Wilson, Gabow, Marks, & Sander, 2008), and miRTarBase (http://mirtarbase.mbc.nctu.edu.tw/php/index.php, Chou et al., 2018). Target predicted by at least three algorithms were selected for the following studies.

### 2.4 Functional analyses

Database for Annotation, Visualization and Integrated Discovery (DAVID; https://david.ncifcrf.gov, Jiao et al., 2012) was employed to perform gene ontology (GO) including cellular component (CC), molecular function (MF), and biological process (BP) analysis and Kyoto Encyclopedia of Genes and Genomes (KEGG) analysis with the purpose of understanding the biological functions of these genes. Parameter *p* < .01 was used as threshold.

### 2.5 Gene expression level validation

The expression level of DEMs and their target was validated at StarBase (Li, Liu, Zhou, Qu, & Yang, 2014).

### 2.6 miRNA‐mRNA regulatory network construction

miRNA‐mRNA regulatory network related to the onset of HCC was visualized using CytoScape V_3.6.0 software (Shannon et al., 2003).

## 3 RESULTS

### 3.1 Identification of DEMs

**Figure 1:**
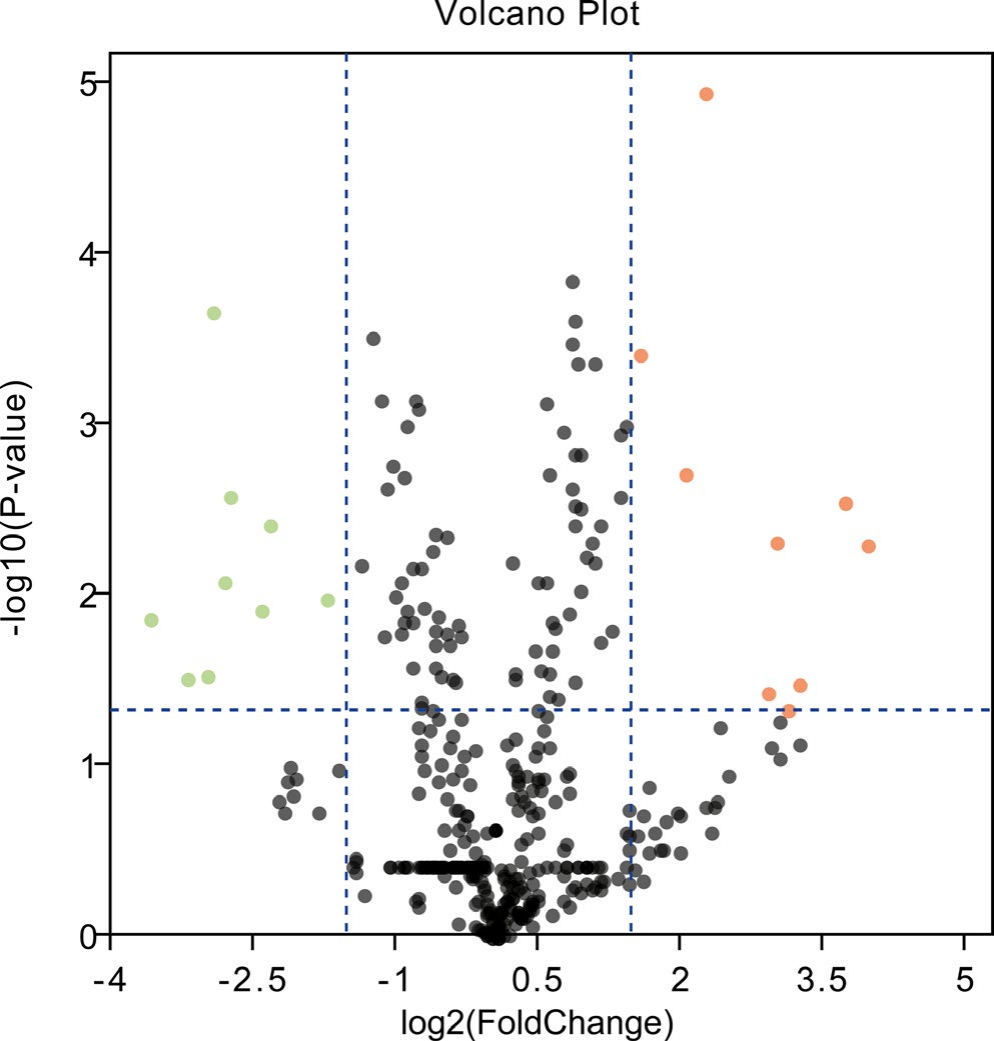
Volcano Plot of differentially expressed miRNAs. Abscissa axis indicates log2 (Fold Change). Vertical axis indicates −log10 (*p* value). Green represents downregulated miRNAs. Red represents upregulated miRNAs. Black indicates no significantly differentially expressed miRNAs. miRNAs, microRNAs

**Figure 2:**
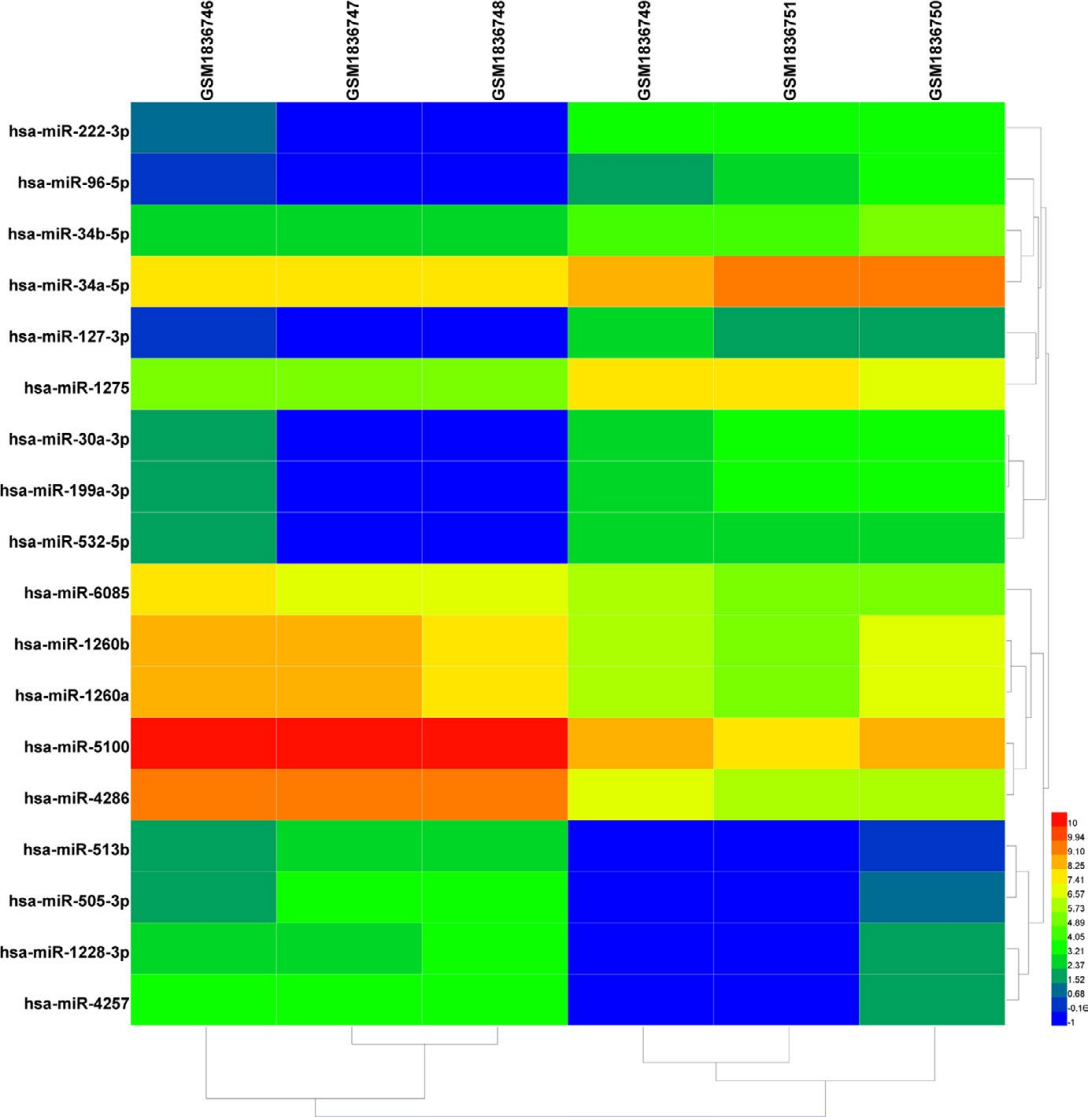
Heat map of differentially expressed miRNAs. Changes in *p* < .05 and |log2 fold‐change| ≥ 1.5 are illustrated by a heat map. miRNAs, microRNAs

As presented in Figure 1, nine upregulated and nine downregulated DEMs were identified in primary human hepatocytes with AFB1 exposure compared to those without. Moreover, heatmap of identified DEMs was presented in Figure 2. We showed that the samples with or without AFB1 treatment were clearly divided into two groups, suggesting the reliability to analyze DEMs in the following experiments.

### 3.2 Target genes of DEMs

Five miRNA target prediction algorithms were employed to analyze potential targets of these identified DEMs. Targets predicted by at least 3 algorithms were selected for followingly analyses. Tables 1 and 2 displayed the targets that contained the most and second‐most binding sites for upregulated and downregulated DEMs, respectively.

**Table 1:** Targets contain the most and second‐most binding site for upregulated DEMs

Target	miRNA list
KMT2A	hsa‐miR‐222‐3p, hsa‐miR‐96‐5p, hsa‐miR‐30a‐3p, hsa‐miR‐199a‐3p
ANKRD52	hsa‐miR‐222‐3p, hsa‐miR‐96‐5p, hsa‐miR‐199a‐3p
NAP1L1	hsa‐miR‐222‐3p, hsa‐miR‐30a‐3p, hsa‐miR‐199a‐3p
NAA25	hsa‐miR‐222‐3p, hsa‐miR‐30a‐3p, hsa‐miR‐199a‐3p
RFX7	hsa‐miR‐222‐3p, hsa‐miR‐30a‐3p, hsa‐miR‐199a‐3p
CTDSPL2	hsa‐miR‐222‐3p, hsa‐miR‐30a‐3p, hsa‐miR‐532‐5p
PPP3R1	hsa‐miR‐222‐3p, hsa‐miR‐96‐5p, hsa‐miR‐30a‐3p
REV1	hsa‐miR‐222‐3p, hsa‐miR‐96‐5p, hsa‐miR‐30a‐3p
BRWD1	hsa‐miR‐222‐3p, hsa‐miR‐96‐5p, hsa‐miR‐30a‐3p
ARF4	hsa‐miR‐222‐3p, hsa‐miR‐96‐5p, hsa‐miR‐30a‐3p
FOXP1	hsa‐miR‐222‐3p, hsa‐miR‐34b‐5p, hsa‐miR‐34a‐5p
SNX4	hsa‐miR‐222‐3p, hsa‐miR‐96‐5p, hsa‐miR‐34a‐5p
MITF	hsa‐miR‐222‐3p, hsa‐miR‐96‐5p, hsa‐miR‐34b‐5p
RNF4	hsa‐miR‐222‐3p, hsa‐miR‐34b‐5p, hsa‐miR‐34a‐5p
PURA	hsa‐miR‐222‐3p, hsa‐miR‐199a‐3p, hsa‐miR‐532‐5p
UBE2J1	hsa‐miR‐222‐3p, hsa‐miR‐30a‐3p, hsa‐miR‐199a‐3p
QKI	hsa‐miR‐222‐3p, hsa‐miR‐30a‐3p, hsa‐miR‐199a‐3p
CAMK2N1	hsa‐miR‐96‐5p, hsa‐miR‐30a‐3p, hsa‐miR‐532‐5p
RARG	hsa‐miR‐96‐5p, hsa‐miR‐30a‐3p, hsa‐miR‐34a‐5p
ZDHHC17	hsa‐miR‐96‐5p, hsa‐miR‐30a‐3p, hsa‐miR‐34a‐5p
ARPP19	hsa‐miR‐96‐5p, hsa‐miR‐532‐5p, hsa‐miR‐1275
VAT1	hsa‐miR‐96‐5p, hsa‐miR‐30a‐3p, hsa‐miR‐34a‐5p
MAPRE1	hsa‐miR‐96‐5p, hsa‐miR‐30a‐3p, hsa‐miR‐199a‐3p
FAM49B	hsa‐miR‐96‐5p, hsa‐miR‐199a‐3p, hsa‐miR‐34b‐5p
ARID4B	hsa‐miR‐30a‐3p, hsa‐miR‐34b‐5p, hsa‐miR‐34a‐5p
SH3PXD2A	hsa‐miR‐30a‐3p, hsa‐miR‐532‐5p, hsa‐miR‐1275
TAOK1	hsa‐miR‐30a‐3p, hsa‐miR‐199a‐3p, hsa‐miR‐1275
NOTCH1	hsa‐miR‐30a‐3p, hsa‐miR‐34b‐5p, hsa‐miR‐34a‐5p

Abbreviation: DEMs, differentially expressed microRNAs.

**Table 2:** Targets contain the most and second‐most binding site for downregulated DEMs

Target	miRNA list
TNRC6B	hsa‐miR‐1260b, hsa‐miR‐513b, hsa‐miR‐4286, hsa‐miR‐1228‐3p, hsa‐miR‐4257
ZFHX4	hsa‐miR‐6085, hsa‐miR‐513b, hsa‐miR‐1228‐3p, hsa‐miR‐4257
POU2F1	hsa‐miR‐1260b, hsa‐miR‐4286, hsa‐miR‐1228‐3p, hsa‐miR‐4257
TFDP2	hsa‐miR‐1260b, hsa‐miR‐5100, hsa‐miR‐4286, hsa‐miR‐1260a
FAM122B	hsa‐miR‐1260b, hsa‐miR‐513b, hsa‐miR‐4286, hsa‐miR‐505‐3p
NUFIP2	hsa‐miR‐1260b, hsa‐miR‐5100, hsa‐miR‐1228‐3p, hsa‐miR‐1260a
FAM222B	hsa‐miR‐6085, hsa‐miR‐5100, hsa‐miR‐4286, hsa‐miR‐1228‐3p
TBL1XR1	hsa‐miR‐5100, hsa‐miR‐513b, hsa‐miR‐4286, hsa‐miR‐4257
CELF1	hsa‐miR‐513b, hsa‐miR‐4286, hsa‐miR‐505‐3p, hsa‐miR‐4257

Abbreviation: DEMs, differentially expressed microRNAs.

### 3.3 Functional analyses of the upregulated or downregulated DEMs

**Figure 3:**
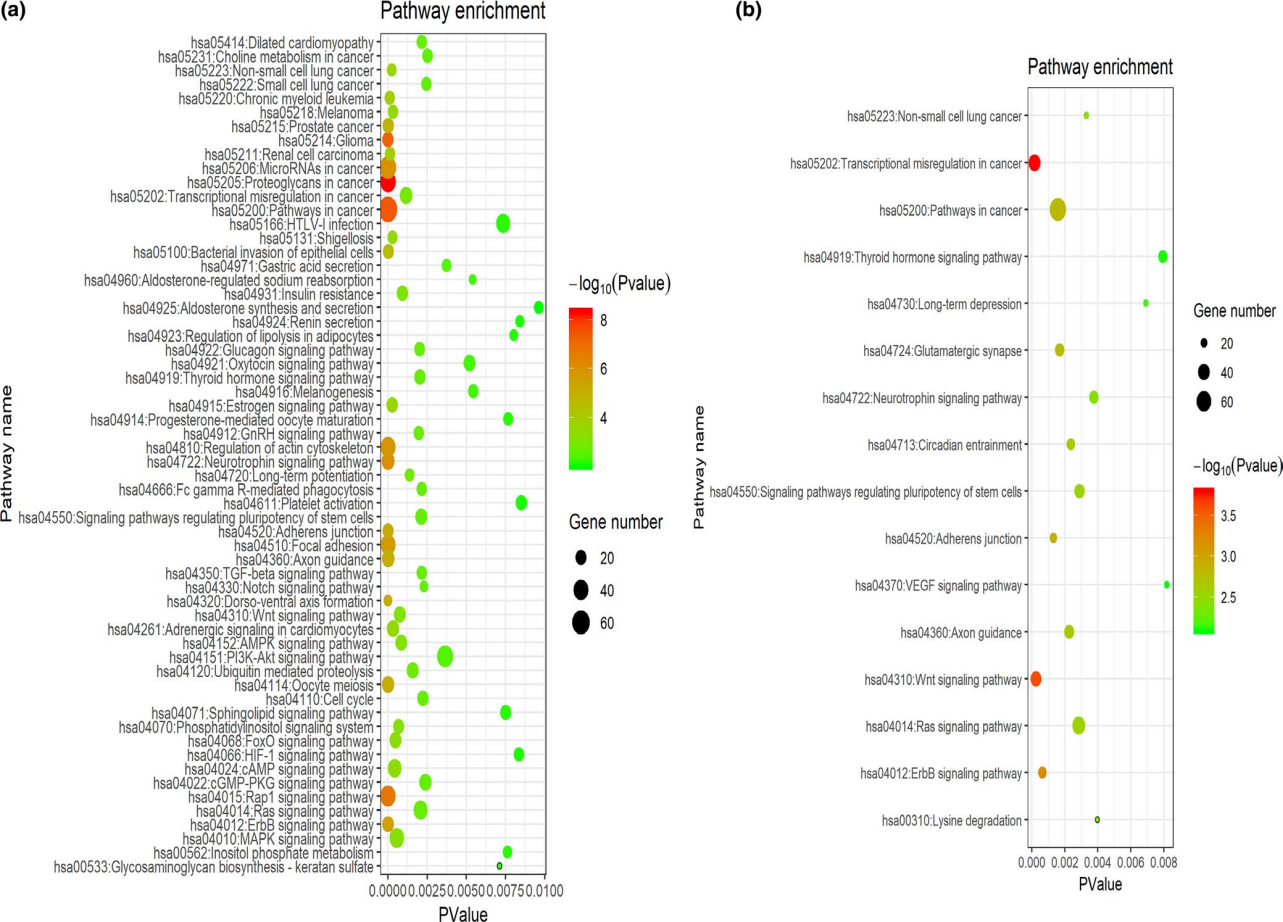
Bubble chart of KEGG pathway enrichment analyses for (a) Upregulated DEMs and (b) downregulated DEMs. Different bubble color represents *p* value. Red indicates strong significance. Bubble size indicates gene numbers enriched in pathway. DEMs, differentially expressed microRNAs; KEGG, Kyoto Encyclopedia of Genes and Genomes

Gene ontology analysis including BP, CC and MF terms for the targets of upregulated or downregulated DEMs were performed to understand the roles of these DEMs. As displayed in Tables 3 and 4, our results revealed that these DEMs have crucial roles in regulating gene expression and cell behaviors. KEGG pathway enrichment analysis in Figure 3a,b revealed that the most enriched pathways were related to cancer development.

**Table 3:** GO analysis of targets of the upregulated DEMs

Category	ID	Description	Count	*p* value
Biological process	GO:0045944	Positive regulation of transcription from RNA polymerase II promoter	174	5.08E‐22
	GO:0000122	Negative regulation of transcription from RNA polymerase II promoter	121	1.66E‐13
	GO:0045893	Positive regulation of transcription, DNA‐templated	94	1.00E‐12
	GO:0006366	Transcription from RNA polymerase II promoter	91	1.22E‐11
	GO:0045892	Negative regulation of transcription, DNA‐templated	80	2.79E‐08
Molecular function	GO:0005515	Protein binding	934	3.94E‐29
	GO:0003700	Transcription factor activity, sequence‐specific DNA binding	142	1.69E‐11
	GO:0001077	Transcriptional activator activity, RNA polymerase II core promoter proximal region sequence‐specific binding	49	6.02E‐09
	GO:0000978	RNA polymerase II core promoter proximal region sequence‐specific DNA binding	64	8.19E‐09
	GO:0043565	Sequence‐specific DNA binding	81	5.53E‐08
Cellular component	GO:0005654	Nucleoplasm	351	1.12E‐19
	GO:0005737	Cytoplasm	563	1.53E‐16
	GO:0005634	Nucleus	579	1.72E‐16
	GO:0005829	Cytosol	372	1.52E‐12
	GO:0016020	Membrane	266	2.97E‐12

Abbreviations: DEMs, differentially expressed microRNAs; GO, gene ontology.

**Table 4:** GO analysis of targets of the downregulated DEMs

Category	ID	Description	Count	*p* value
Biological process	GO:0050789	Regulation of biological process	1751	3.20E‐14
	GO:0032502	Developmental process	971	2.54E‐12
	GO:0065007	Biological regulation	1823	4.59E‐12
	GO:0008152	Metabolic process	1739	1.54E‐09
	GO:0009987	Cellular process	2,326	2.36E‐08
Molecular function	GO:0003700	Transcription factor activity, sequence‐specific DNA binding	211	2.91E‐10
	GO:0005515	Protein binding	1,418	1.46E‐09
	GO:0003677	DNA binding	329	2.10E‐09
	GO:0046872	Metal ion binding	371	5.25E‐06
	GO:0,004,674	Protein serine/threonine kinase activity	87	9.51E‐06
Cellular component	GO:0005654	Nucleoplasm	513	2.50E‐10
	GO:0005634	Nucleus	912	2.98E‐09
	GO:0005737	Cytoplasm	861	6.78E‐07
	GO:0030054	Cell junction	102	6.97E‐06
	GO:0045202	Synapse	47	6.87E‐05

Abbreviations: DEMs, differentially expressed microRNAs; GO, gene ontology.

### 3.4 Validation of DEMs expression in HCC using StarBase

**Figure 4:**
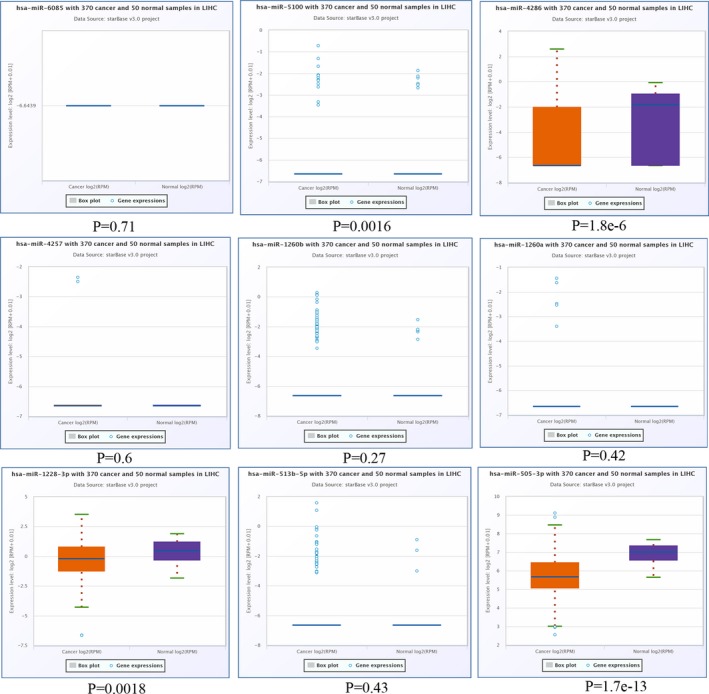
Validation of the expression pattern of downregulated DEMs in StarBase. DEMs, differentially expressed microRNAs

**Figure 5:**
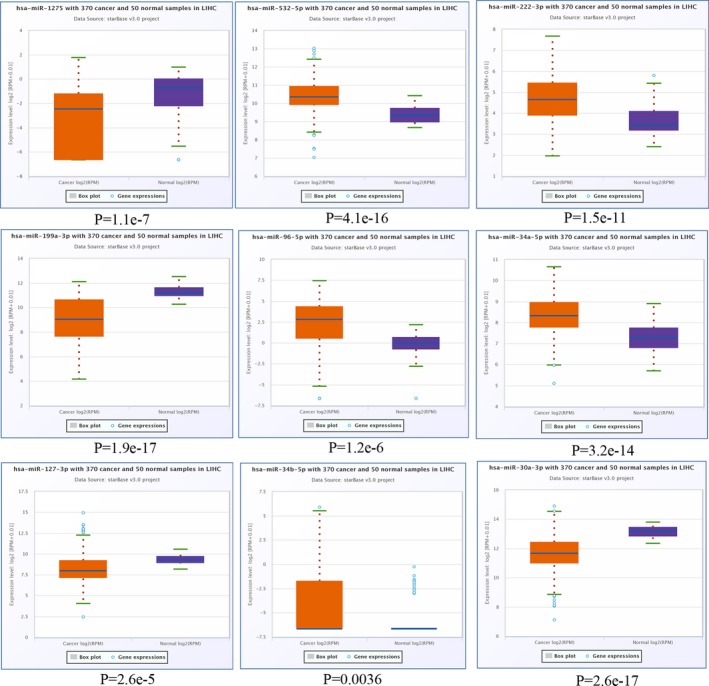
Validation of the expression pattern of upregulated DEMs in StarBase. DEMs, differentially expressed microRNAs

Next, we validated the expression of the DEMs identified in HCC. As presented in Figure 4, the expression pattern of *hsa‐MIR‐505‐3p*, *hsa‐MIR‐1228‐3p*, *hsa‐MIR‐4286*, and *hsa‐MIR‐5100* of the nine downregulated DEMs was similar to the GEO dataset. For the nine upregulated DEMs, we found that all of them exhibited a similar expression trend in HCC as compared with the GEO dataset (Figure 5).

### 3.5 Regulatory network analysis

**Figure 6:**
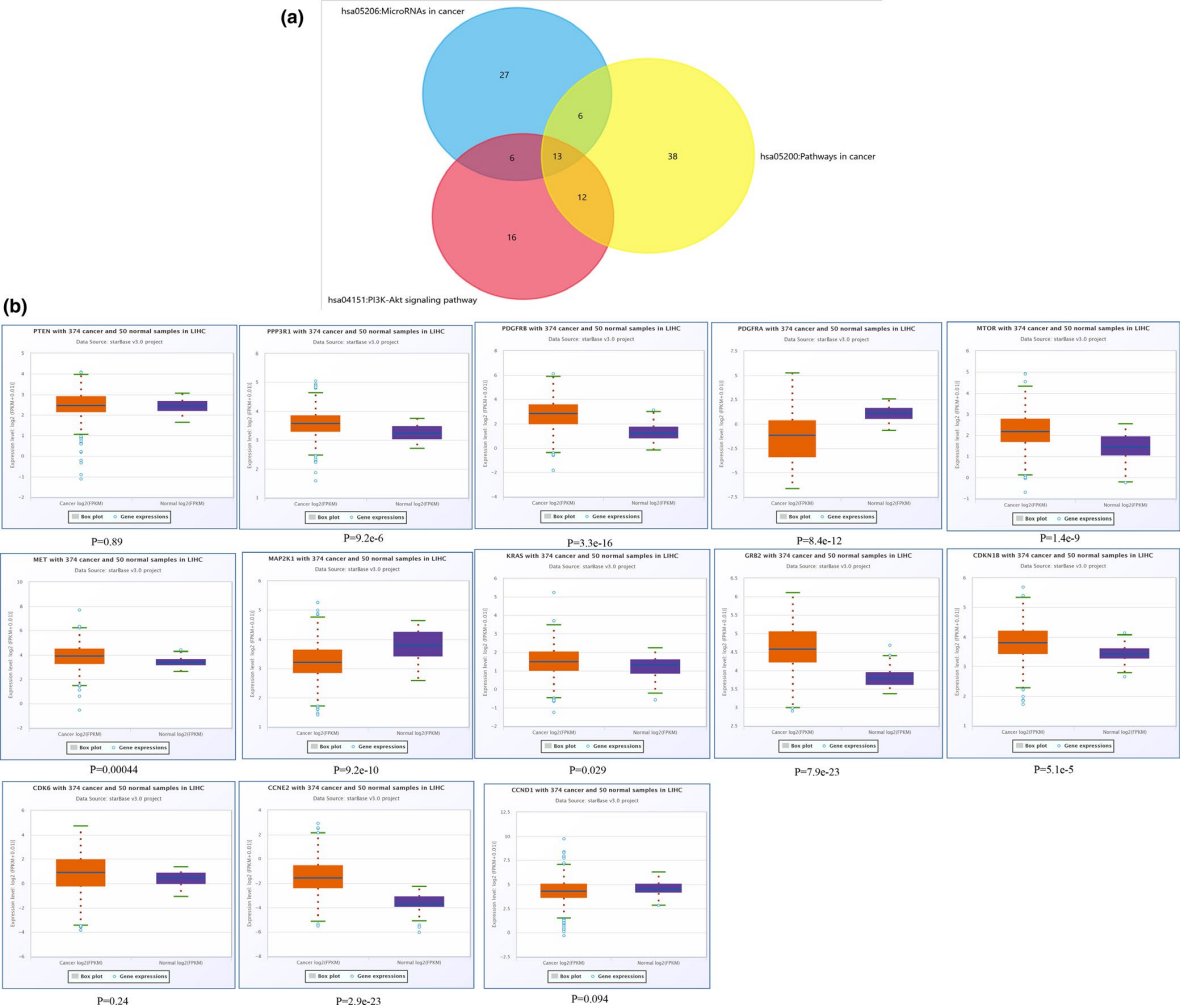
Identification of genes related to HCC progression for upregulated DEMs. (a) Venn diagram indicated intersection of three pathways associated with onset of HCC for the target genes of upregulated DEMs. (b) Validation of the overlapping gene of these three pathways using StarBase. DEMs, differentially expressed microRNAs; HCC, hepatocellular carcinoma

**Figure 7:**
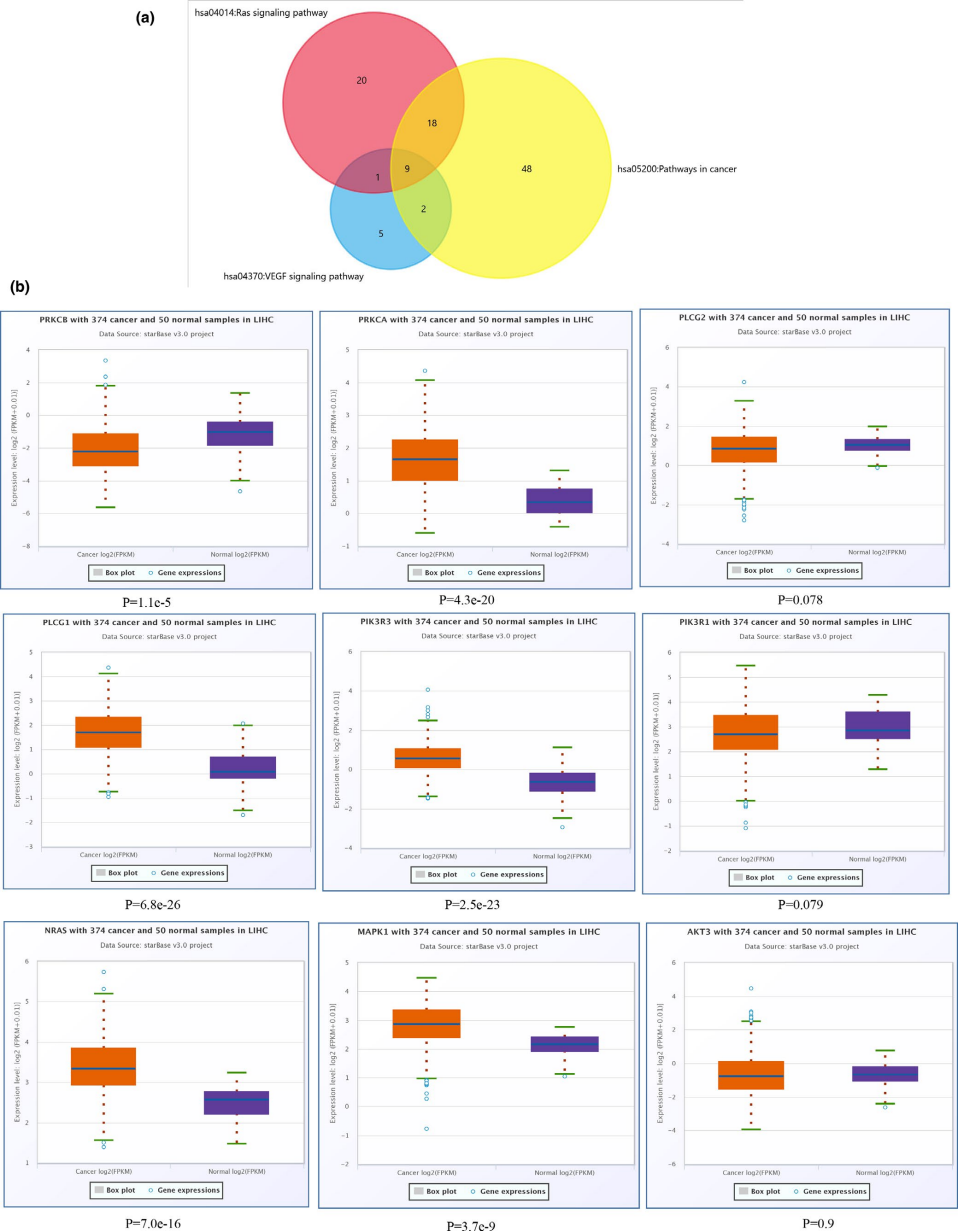
Identification of genes related to HCC progression for downregulated DEMs. (a) Venn diagram indicated intersection of three pathways associated with onset of HCC for the target genes of downregulated DEMs. (b) Validation of the overlapping gene of these three pathways using StarBase. DEMs: differentially expressed microRNAs; HCC, hepatocellular carcinoma

**Figure 8:**
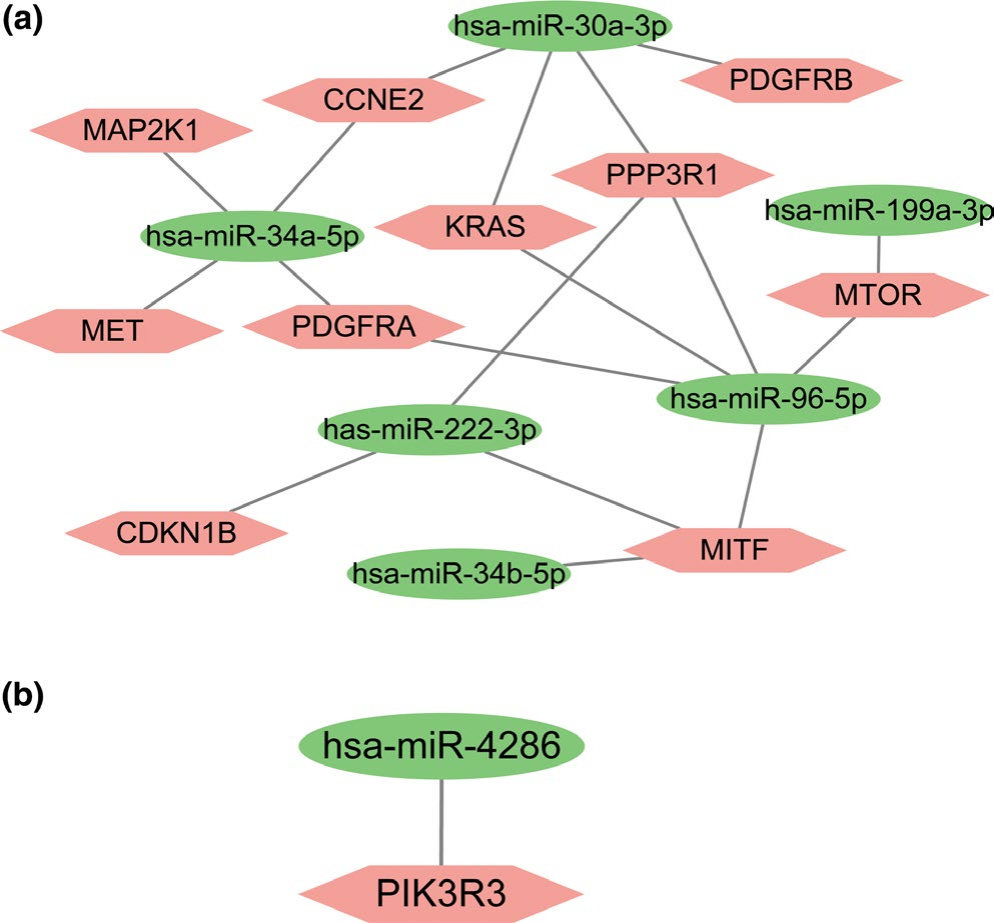
miRNA‐mRNA regulatory network regarding AFB1‐induced onset of HCC. (a) Upregulated DEMs and (b) downregulated DEMs. AFB1, Aflatoxin B1; DEMs, differentially expressed microRNAs; HCC, hepatocellular carcinoma; miRNA, microRNA; mRNA, message RNA

We were interested to identify miRNA‐mRNA networks after AFB1 exposure. Hence, three KEGG pathways related to cancer initiation were selected after pathway enrichment analyses. Three KEGG pathways including miRNAs in cancer, Pathways in cancer, and PI3K‐Akt signaling pathway for upregulated DEMs were selected and 13 overlapping genes were identified for following analyses (Figure 6a). The expression of these 13 genes in HCC was validated in StarBase, and 10 of them were found to be abnormally expressed (Figure 6b). Similarly, the pathways including Pathways in cancer, Ras signaling pathway, and VEGF signaling pathway for downregulated DEMs were analyzed with nine genes identified as shown in Figure 7a. Also, we confirmed the expression of these 9 genes in HCC using Starbase. We found 6 (MAPK1 (176948), NRAS (164790), PIK3R3 (606076), PLCG1 (172420), PRKCA (176960), and PRKCB (176970)) of them were indeed elevated expresion in HCC (Figure 7b). Based on these results, we further established the miRNA‐mRNA regulatory network for upregulated DEMs (Figure 8a) and downregulated DEMs (Figure 8b). As shown in Figure 8a, *hsa‐MIR‐96‐5p* (611606) targeted the largest number of genes in the network. Meanwhile, we found only one downregulated miRNA, *hsa‐MIR‐4286* in Figure 8b.

## 4 DISCUSSION

Aflatoxin B1 is a highly toxic reagent produced by *Aspergillus flavus* and the exposure to AFB1 is reported to account for the initiation of approximately 4%–28% of all HCC cases (Liu & Wu, 2010). Therefore, detection of early fingerprints after AFB1 exposure may help us to prevent the cell development into HCC (Fedeles, Chawanthayatham, Croy, Wogan, & Essigmann, 2017). It was well‐recognized that miRNAs can regulate almose all cellular processes. Therefore, we hypothesized that miRNAs expression may also be altered in AFB1 treated cells. Importantly, studies comprehensively investigating the connection between miRNAs and AFB1 treatment are still lacking.

In this study, a miRNA expression profile dataset that contains data from primary human hepatocytes treated with AFB1 for 5 days and then subjected to 3 days wash‐out period was used to investigate the changed miRNAs after AFB1 withdraw. A total of 18 DEMs including nine upregulated DEMs and nine downregulated DEMs were identified. After targets prediction for these identified DEMs, we analyzed the biological functions using GO annotation and KEGG pathway enrichment methods. We found that the pathways of these genes involved were significantly associated with human cancer progression, which indicated that AFB1 exposure will indeed activate the development of cancers. On the basis of these analyses results, we constructed the miRNA‐mRNA regulatory network to help us understand the mechanisms related to HCC onset after AFB1 exposure. *hsa‐miR‐96‐5p* in the upregulated DEMs associated miRNA‐mRNA network has the largest target numbers. *hsa‐miR‐34a* was previously reported to correlate with liver tumorigenesis after AFB1 exposure (Zhu et al., 2015). Furthermore, *hsa‐miR‐96‐5p*, *hsa‐MIR‐30a‐5p* (612326), *hsa‐MIR‐199a‐3p* (610719), and *hsa‐MIR‐222‐3p* (300569) were previously reported to be associated with HCC progression (Callegari et al., 2018; de Conti et al., 2017; Iwai et al., 2018; Zhang, Liu, Zhang, & Liu, 2017). However, the role of *hsa‐MIR‐34b‐5p* (611374) in HCC has not been reported to date and therefore our study implied that *hsa‐MIR‐34b‐5p* may be related to HCC onset. For the regulatory network constructed by downregulated DEMs, we have noted that the role of *hsa‐MIR‐4286* was not reported to be associated with HCC previously. Therefore, it deserves to be investigated in the future.

This work established miRNA‐mRNA regulatory networks related to the HCC onset by comprehensive bioinformatic analyses. The limitation in this work was that the predicted miRNA‐mRNA link was not experimentally validated, which need further investigations. Hence, future investigations on in vitro cell lines or in vivo animal models are required to validate the prediction results.

In summary, our in‐silico analyses results indicated miRNA‐mRNA connections contributing to the HCC onset after AFB1 exposure. We hope that our results can accelerate the process of tackling the AFB1‐induced onset of HCC.

## CONFLICT OF INTEREST

The authors declare that they have no conflict of interest.
